# Visuospatial perspective-taking of a protagonist during narrative comprehension: the effects of task load and individual differences in visuospatial working memory

**DOI:** 10.3389/fpsyg.2024.1379472

**Published:** 2024-06-12

**Authors:** Asako Hosokawa, Shinji Kitagami

**Affiliations:** ^1^Department of Psychology, Graduate School of Informatics, Nagoya University, Nagoya, Japan; ^2^Japan Society for the Promotion of Science, Tokyo, Japan

**Keywords:** cognitive psychology, sentence comprehension, narrative comprehension, visuospatial working memory, visuospatial perspective-taking

## Abstract

**Introduction:**

This study examined whether visuospatial perspective uses the character perspective during narrative comprehension.

**Method:**

Participants read narrative stimuli depicting the spatial positional relationships between characters and objects and judged whether the objects were on the left or right from the character's perspective. We manipulated whether the spatial positional relationships between characters depicted in the narrative stimuli resulted in a visuospatial perspective. We hypothesized that the high-load perspective-taking condition would indicate longer reaction times compared to the low-load perspective-taking condition, as shifting perspectives between characters in the high-load condition require more time for visuospatial perspective-taking.

**Results:**

As predicted, the reaction time was longer for high-load perspective-taking than for low-load perspective-taking.

**Discussion:**

During narrative comprehension, the reaction time for visuospatial perspective-taking must move virtually within the representation from the main character's perspective to that of another character. Visuospatial perspective-taking is involved in narrative comprehension.

## 1 Introduction

When you read a narrative, you may have the experience of immersing yourself in the narrative and feeling as if you have entered that world. When comprehending a text, it is suggested that the reader is experiencing a mental simulation of an imagined thing or situation as if they were experiencing the real thing (Fincher-Kiefer, 2019). For example, just a description of someone eating a hamburger would conjure up images of the hamburger, fries, and the restaurant setting. Mental representations constructed during text comprehension contain perceptual information, such as visuospatial images. Zwaan et al. (2002) presented participants with sentences implying shapes, such as “The ranger saw the eagle in the sky,” followed by images of either a spread-winged eagle (matching condition) or a perched eagle with folded wings (mismatching condition). The results indicated that participants exhibited significantly faster reaction times when the images matched the sentences. These findings indicate that readers construct perceptual simulations during language comprehension, which facilitates the processing of congruent visual information. Additionally, brain regions involved in visual imagery are more activated when processing imagery-rich texts (Just et al., 2004), including perceptual information derived from readers' experiences and knowledge (Zwaan, 2004).

The perceptual availability hypothesis states that perceptual processing is generated as if the reader is experiencing the constructed narrative world during narrative comprehension (Horton and Rapp, 2003). To test this hypothesis, Horton and Rapp (2003) examined narrative events that affected the perceptual perspective of protagonists, using a task where participants judged whether an object was mentioned in the preceding sentence. They found that reaction times were slower when the sentence described a situation where the object was not visible to the protagonist (e.g., because it was “shielded”) compared to when the object was visible to the protagonist. The perceptual availability hypothesis is an important finding that indicates readers could mentally simulate the world in reading. Many studies suggested Visuospatial (VS) representations based on the protagonist's perspective reflect the actual VS perspective (e.g., Borghi et al., 2004; Yaxley and Zwaan, 2007; Horchak and Garrido, 2020). However, these studies used only single-sentence stimuli. At present, the perceptual availability hypothesis has not been tested further because long narrative texts are complex and difficult to control as experimental stimuli. Therefore, this study aims to examine perceptual availability hypothesis by employing multi-sentence texts.

To extend the perceptual availability hypothesis, we focused on spatial cognition studies of VS perspective-taking. In spatial cognition research, VS perspective-taking requires changing perspectives from one's position to that of others (Zacks and Michelon, 2005). VS perspective-taking involves an embodied movement in which the self's body is moved virtually to the position from which the other's perspective is to be taken. Kessler and Thomson (2010) suggested that the greater the angle between the position of the self and the target, the longer the embodied movement time. Therefore, a longer time is required for VS perspective-taking. People transform their body schema into the perspective of the other person and place their own body in that position (Erle and Topolinski, 2017). Consequently, embodied movement toward the other person facilitates VS perspective-taking, while movement away makes it more difficult. In recent years, researchers have hypothesized that there is a shared basis between mental perspective-taking related to empathy and spatial perspective-taking (Erle and Topolinski, 2015, 2017). In the process of mental perspective-taking during narrative comprehension, there is a possibility that taking the perspective of a character enhances the reader's understanding of that character's emotional state. In other words, a richer mental simulation of the protagonist's emotional state is formed when the reader takes on the protagonist's perspective.

Concerning narrative comprehension, Horton and Rapp (2003) demonstrated that VS representations are constructed to reflect whether an object is visible from the protagonist's perspective in reading. This study investigated VS processing during narrative comprehension based on the perceptual availability hypothesis. According to this hypothesis, readers construct VS mental representations during reading as if they were perceiving the situation. As part of this representation construction, readers adopt the characters' perspectives. Importantly, VS perspective-taking is based on textual information. Determining whether the ability to shift to the perspectives of several characters leads to a more multidimensional understanding of the narrative would clarify if adopting multiple perspective facilitates comprehension. Therefore, by verifying whether VS perspective shifts occur during narrative comprehension, this study examines the validity of the perceptual availability hypothesis to broaden its scope.

Further, VS information such as perspective is likely to depend on individual differences between readers. Readers' visuospatial working memory (VSWM) capacity is related to the activation of VS processing in reading (Vermeulen et al., 2008). VS processing resources are involved and constructing representations of its content during narrative comprehension (Fincher-Kiefer, 2001; Fincher-Kiefer and D'Agostino, 2004). Gillioz et al. (2012) reported that individuals with larger VSWM capacity are more likely to find VS information contained in a narrative more easily, suggesting that individual differences in VSWM capacity may be involved in the activation of perceptual processing. In addition, we controlled for individual differences in mental perspective-taking ability using the Interpersonal Reactivity Index (IRI; Davis, 1983). The IRI, a quantitative measure of dispositional empathy in separate dimensions, investigates the effects of experimental VS perspective-taking manipulation by accounting for the mental perspective-taking variability of related VS perspective-taking.

Therefore, this study examined whether VS perspective-taking from the character's perspective arises by measuring the time required for participants to judge the relative positions of objects from the character's perspective during narrative comprehension. To test this hypothesis, participants read narrative texts in which the spatial positional relationships between characters were manipulated under conditions of perspective-taking. Specifically, the relative positional relationships between characters were set at two levels: next to or opposing. The difficulty of perspective-taking was thus manipulated. Subsequently, the time required for perspective-taking was measured to analyze whether it takes more time as the angle between characters increases. This allowed us to examine whether participants adopted the character's perspective during narrative comprehension. If VS perspective-taking arises from the character's perspective during narrative comprehension, we predicted that perspective-taking would take more time under high-load perspective-taking conditions than low-load perspective-taking conditions.

Another focus of this study was to examine whether individual differences affect perspective-taking manipulation. First, it is conceivable that individual differences in VSWM capacity may influence the manipulation strategies of VS perspective-taking. Additionally, as mentioned earlier, mental perspective-taking ability may be related to the process of VS perspective-taking. Therefore, we examined the influence of individual differences in VSWM capacity while controlling for individual differences in mental perspective-taking ability. We predicted an interaction between performances on the VS perspective-taking task and individual differences in VSWM.

## 2 Methods

### 2.1 Participants

Fifty-one university students in Japan (21 men and 30 women; mean age = 22.16 ± 3.39 years) participated. All were native Japanese speakers. The sample size was determined by referring to previous studies on narrative comprehension (e.g., Komeda et al., 2013; Magliano et al., 2016), which have established a standard sample size in this research area. Participants were recruited from March 3 to April 30, 2023, within the university. Written informed consent was obtained from all participants before the commencement of the experiment. The consent form provided information regarding anonymity, confidentiality, and the right to voluntarily withdraw at any time. This study received ethical approval from the University's Research Ethics Committee (No. NUPSY-230901-I-01).

### 2.2 Stimuli, tests, and index

#### 2.2.1 Narrative stimuli

The first author constructed eight narrative stimuli, which included three characters (one protagonist and two supporting characters) and two objects. Each narrative contained seven sentences. The composition of each narrative stimulus was as follows: First, the initial two sentences depicted the three characters and their spatial relationship; one character was positioned next to the protagonist and the other was positioned across from the protagonist. The next three sentences introduced two objects and described their spatial relationship. The objects were positioned side-by-side between the three characters. The final two sentences led the participant to take the viewpoint of the protagonist by portraying the protagonist's emotions and actions, concluding the narrative. In addition, the sexes of the characters were separated to match those of the participants and characters. Incidentally, the only difference was in the name of the characters; all the content was the same. Samples used in the experiment are as follows: “*Kenta sat at a table in the restaurant and waited for friends./After a while, Takeshi and Yusuke came over. Takeshi sat opposite Kenta and Yusuke sat next to Kenta./While they were talking, the waiter brought the French fries that Kenta had ordered./The waiter placed the fries in the middle of the table./He then placed the salad right next to it./Takeshi and Yusuke looked at the menu and then ordered something else./Kenta asked the waiter as he passed by*.”

Before the experiment, a pilot study validated the appropriateness of the narrative stimuli with 12 participants (eight men and four women, mean age = 22.08 years), examining whether they imagined the spatial positions of the characters and perceived differences in liking toward the characters. Finally, eight stimuli with no differences in liking ratings were adopted for the main experiment.

#### 2.2.2 Visuospatial perspective-taking task

The VS perspective-taking task was presented immediately after reading the narrative stimuli. Following the narrative stimulus, participants were presented with a VS perspective-taking task, such as: “*From Takashi's perspective, where is the salad located? Right or left?*” The VS perspective-taking task will be discussed in further detail in the subsequent section. The stimuli for the VS perspective-taking task consisted of questions asking whether objects in the narrative were positioned to the left or right from the character's perspective. We manipulated differences in processing load for VS perspective-taking by combining objects and characters; specifically, determining the position of objects from the viewpoint of a character facing the protagonist required perspective transformations to correctly solve the task, creating high cognitive demand (Kessler and Thomson, 2010). Contrastingly, for a character positioned next to the protagonist, embodiment transformations into the target's perspective were unnecessary, allowing judging of object positional relationships from the protagonist's position with a low processing load. This was controlled by counterbalancing the arrangement of objects and characters.

#### 2.2.3 Visuospatial working memory capacity test

A VSWM capacity test was conducted to measure participants' VSWM capacity. This task comprised five trials in total. The visual pattern stimuli were displayed on a white computer screen background within a 4 x 4 matrix with black dots positioned inside the grid spaces. The first trial had four dots placed, and the number of dots increased in proportion to the trial number so that the final fifth trial had eight dots placed. Additionally, for all trials regardless of the task, the placement of the visual patterns was made to differ entirely. VS pattern stimuli were first presented for 1,000 ms. Thereafter, participants were asked to read a sequence of numbers aloud to prevent them from verbally remembering the sequence of dots. After 10 s, the screen switched to one that instructed participants to reconstruct the VS pattern stimulus. Once reconstruction was completed, participants could click on the screen at their own pace to proceed to the next trial. The correct response rate was calculated by using the number of dots presented in each trial as the denominator and the number of correct responses as the numerator. The distribution of participants' VSWM capacity is shown in the Supplementary material.

#### 2.2.4 Interpersonal Reactivity Index

The 28-item Japanese version of the IRI (Davis, 1980) was adapted by Himichi et al. (2017). It measures individual differences in mental perspective-taking. It is measured with a five-point Likert scale, including “empathic concern,” “personal distress,” “perspective-taking,” and “fantasy.” Of these, mental perspective-taking is the ability to consider situations from another person's point of view and infer emotions (Davis, 1983). Perspective-taking has been linked to VS perspective-taking (Erle and Topolinski, 2015). Individual differences in participants' mental perspective-taking could affect their performance on VS perspective-taking tasks. Accordingly, perspective-taking as measured by the IRI perspective-taking subscale was included as an additional predictor in the linear mixed effects models.

### 2.3 Procedure

The experiment comprised three tasks: a VS perspective-taking task, responding to an empathy questionnaire, and a VSWM capacity test. First, a VS perspective-taking task was conducted. The experiment comprised three practice trials and 16 main trials. Out of the 16 critical trials, four trials featured a high-load perspective-taking manipulation and the other four trials had a low-load perspective-taking manipulation. The remaining eight trials served as filler trials.

After reading the on-screen instructions, they clicked the “ready” button to begin the task when they were prepared. In the first part, a narrative stimulus was presented, with each sentence displayed for 7 s. Participants were instructed to read silently to understand. Immediately after the narrative presentation, the task shifted to the VS perspective-taking part. Participants were instructed to respond as quickly and accurately as possible during the part of the trial. VS perspective-taking task began after the narrative stimulus ended and after 500 ms of fixation screen. If participant thought the object was on the left side of the target, they pressed the “C” key. If they thought it was on the right side, they pressed the “M” key. After participants completed the VS perspective-taking task, they completed the IRI and the VSWM capacity test on the screen. Finally, after confirming the participants' age, native language, and ability to predict the purpose of the experiment, the experimenter explained the study content and the experiment was completed. The experiment lasted for ~30 min.

### 2.4 Data analysis

As described in the Methods section, sample size was set based on prior work in the field. However, a *post-hoc* power analysis indicated an achieved power of 0.98, given the sample size of 48, suggesting adequate statistical power was obtained. Therefore, this study can be considered to has a sufficient sample size both in principle and in terms of power analysis.

Analysis was conducted using linear mixed-effects models in R software (R Core Team, 2021). The models included fixed effects of perspective condition, VSWM capacity, and their interaction. Random intercepts for participants, items, and IRI perspective-taking scores were also modeled. Likelihood ratio tests were used to select the best-fitting model. Coding employed for the perspective condition utilizes (−1, 1) sum coding, where (−1) represents the low-load perspective-taking condition and (1) represents the high-load perspective-taking condition.

The random factors included participants, items, and participants' IRI perspective-taking scores to perform linear mixed modeling analysis.^1^ We directly modeled VSWM capacity as a fixed effect alongside the experimental conditions. This enabled us to estimate the causal influence of VSWM on the perspective-taking process in narrative comprehension. Moreover, we accounted for individual differences in empathy, as measured by mental perspective-taking in IRI scores, in the variable effects portion.

Model selection was performed through the following procedure. First, the maximal model that converged, including random intercepts for participants, stimuli, and individual differences in mental perspective taking, as well as their random slope, was constructed. Then, models eliminating random slope terms were built incrementally, removing less influential terms first following Jaeger (2009). Likelihood ratio tests were conducted sequentially between the more intricate model relative to each parsimonious model for model comparison and selection balances model complexity and goodness-of-fit. The model best accounting for the data while retaining interpretability was chosen based on likelihood ratio test results.

## 3 Results

### 3.1 Reaction time

Before analysis, 153 false response trials and nine trials with reaction times >±2.5 *SD* were excluded from the analysis. First, we conducted a Shapiro-Wilk test to assess the normality of the reaction time data. The results indicated that the reaction time data didn't conform to a normal distribution (*W* = 0.96, *p* < 0.01), leading to the rejection of the null hypothesis. Hence, we applied a square-root transformation to the reaction time data and conducted a Shapiro-Wilk test again, which revealed that the transformation improved the normality of the data (*W* = 0.99, *p* = 0.29).

The main dependent variable in this study was participants' reaction times to judge the relative positions of objects from the perspective of characters. Longer reaction times were interpreted as reflecting more effortful perspective-taking processes.

Linear mixed model analysis revealed a main effect for the VS perspective [*Estimate* = 2,814.99, *SE* = 1,212.40, *df* = 202.37, *t* = 2.32, *p* = 0.02, $\eta_{p}^{2}$ = 0.48], but no main effect for VSWM capacity [*Estimate* = −165.57, *SE* = 1,277.10, *df* = 44.33, *t* = 0.13, *p* = 0.90, $\eta_{p}^{2}$ = 0.00]. This confirms that response times were significantly lower for low-load perspective and higher, while participants with higher or lower memory capacity did not differ in their response time. Moreover, the interaction of the VS perspective condition and VSWM capacity was significant [*Estimate* = −3,207.31, *SE* = 1,521.81, *df* = 203.09, *t* = 2.10, *p* = 0.04, $\eta_{p}^{2}$ = 0.29]. A simple slope test revealed that reaction times in the high-load perspective-taking condition were reliably longer than those in the low-load perspective-taking condition in VSWM −1 *SD* [*Estimate* = 718.19, *SE* = 283.64, *t* = 2.53, *p* = 0.01], but there was not a significant difference between the high-load perspective-taking condition and low-load perspective-taking condition in VSWM +1 *SD* [*Estimate* = −138.67, *SE* = 287.11, *t* = 0.48, *p* = 0.63] (Table 1). Thus, response times were significantly longer for the high-load perspective-taking condition than for the low-load perspective-taking condition, especially for participants with lower VSWM capacity. This suggests that individuals with lower visuospatial working memory capacity may have more difficulty with perspective-taking under high cognitive load.

**Table 1:** Fixed effects estimates for reaction time.

	**Reaction time**				
**Effect**	**Estimate**	**SE**	***df***	***t***	***p***
Intercept	63.33	7.84	44.16	8.08	0.001
Visuospatial perspective-taking condition	10.84	4.60	202.21	2.36	0.02
Visuospatial working memory capacity	−0.11	9.79	44.49	−0.01	0.99
Perspective-taking × visuospatial working memory capacity	−12.47	5.78	202.92	−2.16	0.03

Reaction times are presented in milliseconds (ms). Data have been preprocessed as described in the Data analysis subsection of the Methods section, including the exclusion of false response trials and outliers, and the application of a square-root transformation to improve normality.

### 3.2 Correct response rate

linear mixed model analysis revealed a non-significant main effect of VS perspective-taking condition [*Estimate* = −1.24, *SE* = 0.70, *z* = −1.78, *p* = 0.08, *OR* = 0.29]. However, no main effect for VSWM capacity [*Estimate* = −0.93, *SE* = 0.85, *z* = −1.10, *p* = 0.27, *OR* = 0.39]. Moreover, the interaction between VS perspective-taking and VSWM capacity was significant [*Estimate* = 1.83, *SE* = 0.81, *z* = 2.25, *p* = 0.02, *OR* = 6.21]. A simple slope test revealed no significant differences in correct response rates between the perspective-taking and low-load perspective-taking conditions at either −1 *SD* (*Estimate* = 0.47, *SE* = 0.28, *z* = 1.65, *p* = 0.10) or +1 *SD* (*Estimate* = −0.03, *SE* = 0.28, *z* = −0.10, *p* = 0.92) of VSWM (Table 2).

**Table 2:** Fixed effects estimates for correct reaction rate.

	**Correct reaction rate**			
**Effect**	**Estimate**	**SE**	***z***	***p***
Intercept	1.28	0.73	1.75	0.08
Visuospatial perspective-taking condition	−1.24	0.70	−1.78	0.08
Visuospatial working memory capacity	−0.93	0.85	−1.10	0.27
Perspective-taking × visuospatial working memory capacity	1.83	0.81	2.25	0.02

Correct reaction rates using a generalized linear mixed model. Random effects have been factored in for participants, stimuli and mental perspective taking.

## 4 Discussion

This study examined to examine if VS perspective-taking of characters occurs and whether it is influenced by individual differences in VSWM capacity during narrative comprehension. Specially, we examined whether participants read narrative stimuli in which the spatial positioning of characters and objects was described, and judged whether two objects were left or right from the character's perspective. Consequently, reaction times were significantly longer in the high-load perspective-taking condition than in the low-load perspective-taking condition. This suggests that embodied motion is generated from the perspective of one character within a visual representation of the perspective of another character. In addition, VS perspective-taking arose despite controlling for individual differences in mental perspective-taking, as measured by the IRI. Our results are consistent with our first hypothesis, indicating that participants represent the characters' VS perspectives regardless of their ability to engage in mental perspective-taking during narrative comprehension.

Consequently, the most important finding of this study is consistent with and extends Horton and Rapp's (2003) perceptual availability hypothesis. Moreover, this result demonstrates embodied movement within the representation of the character's perspective in reading, supporting previous research in the field of spatial cognition (Kessler and Thomson, 2010). Notably, understanding VS perspective-taking may involve comprehending how the world is represented from the character's perspective. This is, based on both visual and textual information. The Japanese language, compared to languages such as English, is characterized by less frequent use of prepositions and more frequent use of particles to express spatial relationships (Oka, 2007). Thus, Japanese readers may need to allocate more cognitive resources to infer the spatial relationships between characters. Despite such linguistic characteristics, this study suggests the possibility that VS perspective-taking occurs in Japanese narrative comprehension.

Participants with a lower VSWM capacity took more time to develop perspectives. This result provides evidence consistent with our second hypothesis, which is that individual differences in VSWM would also indicate different performances in VS perspective-taking. Although the interaction between VS perspective-taking and VSWM capacity on accuracy was significant, a simple slope test showed a non-significant trend. Participants with lower VSWM capacity needed more time to form perspectives, and individual differences in VSWM would also indicate different performance in VS perspective-taking. The significant interaction between VS perspective-taking and VSWM capacity on accuracy suggests that VSWM plays a role in the accuracy of perspective-taking. However, the non-significance in the simple slope test indicates that the relationship between VSWM and perspective-taking accuracy is not significant. Comparing the current results of with Gillioz et al. (2012), it is similar that a lower VSWM capacity requires more time for the elaboration of spatial information, highlighting the role of VSWM in processing spatial information efficiently. However, the lack of difference in accuracy based on VSWM size in our study contrasts with Gillioz et al. (2012). This discrepancy may be attributed to task differences. While Gillioz et al. (2012) focused on spatial information extraction, This study involved the additional cognitive demands of maintaining and switching perspectives. This suggests that individuals can compensate for lower VSWM capacity by investing more cognitive resources to achieve comparable levels of elaboration although VSWM differences influence the time required to simulate a situation. The cognitive costs associated with perspective-taking may be higher for lower VSWM capacity. However, they can still perform the task accurately by allocating more cognitive effort.

This study had some methological limitations. First, we assumed that participants adopted the protagonist's perspective when comprehending the narrative stimuli. However, the reliability of this manipulation was questionable. It should add conditions in which object positions are judged from the perspective of the protagonist, or have participants describe the narrative perspective they adopted after the experiment. Moreover, Using first-person pronouns such as you or I to refer to the protagonist would enable clearer determination of the initial narrative perspective, make perspective easier, and reduce the extraneous cognitive load of memorizing characters' names. Second, the narrative stimuli automatically progressed every seven seconds, preventing rereading of previous sentences. This may introduce confounding factors related to individual differences in memory abilities and strategies. It should investigate memory-related variables by comparing conditions that collect data on both VS and verbal working memory capacities. Third, the non-standardized VS memory task utilized to assess VSWM capacity lacks sufficient evidence of validity, despite being designed to assess core VSWM skills and having been used in previous studies (e.g., Fincher-Kiefer, 2001; Fincher-Kiefer and D'Agostino, 2004; Suto and Hyodo, 2006). To substantiate the veracity of these findings, it is imperative to replicate them using a standardized VSWM test.

Future research should aim to clarify the specific processes in VS perspective-taking during narrative comprehension and their links to individual differences in working memory. This could involve using more fine-grained measures of VSWM and manipulating cognitive load. Further, investigating the linguistic aspects of perspective-taking, such as mental state verb production (Neitzel and Penke, 2021), could yield a more comprehensive understanding of perspective-taking in narrative comprehension. Based on the perceptual availability hypothesis, the relationship between VS and mental perspective-taking (Erle and Topolinski, 2017) may also be reflected during narrative comprehension. By pursuing these research directions, we can develop a more comprehensive understanding of how individual differences in cognitive abilities and the interaction with VS and linguistic perspective-taking contribute to narrative experience.

## 5 Conclusion

In conclusion, this study suggests that readers construct VS representations based on characters' perspectives and individual differences in VSWM capacity may influence during narrative comprehension. Our findings contribute to the evidence supporting that readers construct embodied simulations constrained by the perceptual availability hypothesis and highlight the influence of individual differences in VSWM during narrative comprehension.

## Data availability statement

The raw data supporting the conclusions of this article will be made available by the authors, without undue reservation.

## Ethics statement

The studies involving humans were approved by the Nagoya University's Research Ethics Committee. The studies were conducted in accordance with the local legislation and institutional requirements. The participants provided their written informed consent to participate in this study. Written informed consent was obtained from the individual(s) for the publication of any potentially identifiable images or data included in this article.

## Author contributions

AH: Conceptualization, Data curation, Formal analysis, Funding acquisition, Investigation, Methodology, Project administration, Resources, Software, Validation, Visualization, Writing – original draft. SK: Supervision, Writing – review & editing.

## Footnotes

^1^Additional analyses showed a trend for IRI's PT subscale negatively influencing RTs (β = −75.46, *p* = 0.13), although model fit was low.
