# Supplementary material for: Visuospatial perspective-taking of a protagonist during narrative comprehension: the effects of task load and individual differences in visuospatial working memory
Source: Front Psychol. 2024 Jun 12;15:1379472. doi: 10.3389/fpsyg.2024.1379472 (PMC11199894; doi:10.3389/fpsyg.2024.1379472)

Supplementary Material

Visuospatial perspective-taking of a protagonist during narrative comprehension

Asako Hosokawa, Shinji Kitagami

*** Correspondence:**

Asako Hosokawa
[hskwask1512@gmail.com](mailto:hskwask1512@gmail.com)

# Supplementary Data

**Figure1**

*Distribution of Visuospatial Working memory Capacity*
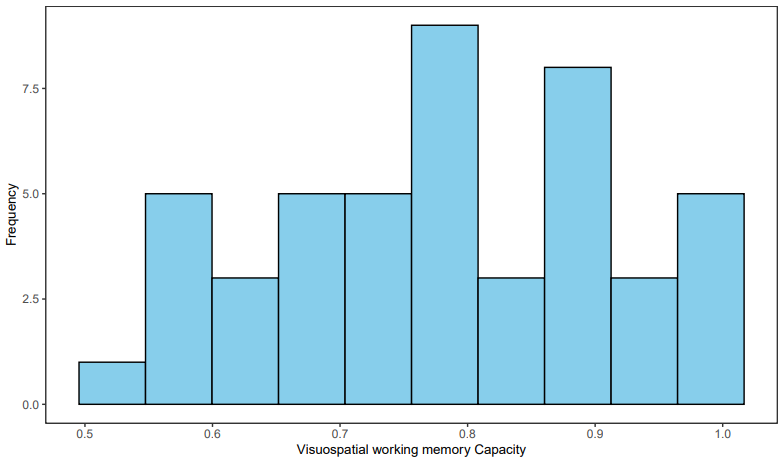

Supplement: Supplementary file 1 [file Table_1.DOCX]
